# Facial asymmetry: a case report of localized linear scleroderma patient with muscular strain and spasm

**DOI:** 10.1186/s40902-015-0029-x

**Published:** 2015-09-16

**Authors:** Jae-Hyung Kim, Suck-Chul Lee, Chul-Hoon Kim, Bok-Joo Kim

**Affiliations:** Department of Oral and Maxillofacial Surgery, Dong-a University Hospital, Daesingonhwon-ro26, Seo-gu, Busan, 602-715 Korea

**Keywords:** Facial asymmetry, Scleroderma, Parry-Romberg syndrome (PRS), Soft-tissue facial asymmetry

## Abstract

Facial asymmetry is found in patients with or without cosmetic facial alterations. Some patients have facial asymmetry that manifests underlying skeletal problems, while others have only limited soft-tissue facial asymmetry. Orthognathic surgery brings about a dermatic change, as soft tissue covers underlying bones. Limited soft-tissue asymmetry, meanwhile, is difficult to correct. The treatment modalities for the creation or restoration of an esthetically pleasing appearance were autogenous fat grafts, cartilage graft, and silicon injections.

A young female patient had right-side facial asymmetry. The clinical assessment involved visual inspection of the face and palpation to differentiate soft tissue and bone. Although the extra-oral examination found facial asymmetry with skin atrophy, the radiographic findings revealed no mandibular atrophy or deviation. She was diagnosed as localized scleroderma with muscle spasm.

In conclusion, facial asymmetry patients with skeletal asymmetry can be esthetically satisfied by orthognathic surgery; however, facial atrophy patients with skin or subdermal tissue contraction need treatment by cosmetic dermatological surgery and orthodontic correction.

## Background

As patients become more aware of and concerned about facial esthetics, so too, they grow more self-conscious about facial asymmetry and more desirous of correcting it. Although most faces show some degree of mild facial asymmetry, not all cases require surgical correction. Facial asymmetry is found in patients with or without cosmetic facial alterations. Some patients have facial asymmetry that manifests underlying skeletal problems, while others have only limited soft-tissue facial asymmetry. Resolution of facial asymmetry, in any case, is becoming an important goal of orthodontic treatment and orthognathic surgery.

Facial asymmetry prevalence rates range between 21 and 85 % [1]. The etiology of facial asymmetry among diverse patients is still unknown, and indeed, its causes are multivariate. Bishara SE et al. reported on the many and various etiologic factors that are implicated in genetic or congenital deformities (cleft lip and palate, condyle hypoplasia, hemifacial microsomia, etc.), developmental asymmetry (arising during growth), and acquired, environmental asymmetry (habits, disease, trauma, etc.) [2]. Limited soft-tissue facial asymmetry, otherwise known as skeletal facial asymmetry, is rare. Its differential diagnosis includes post-traumatic scarring, facial atrophy known as Parry-Romberg syndrome (PRS), and linear scleroderma [3].

Patients with facial asymmetry have not only problems of facial skeletal deformity but also esthetic and morphologic problems related to skin atrophy. To obtain the best resolution, patient diagnosis, treatment planning, and result assessment should be based on accurate skeletal as well as morphological analysis and measurement. To those ends, three-dimensional models, traditional radiologic procedures, and soft-tissue examination are employed.

In cases of soft-tissue facial asymmetry without skeletal deformities, some pathological conditions are found. In 1891, C. J. Nixon reported on localized scleroderma (LS) patients with the rare clinical feature hemiatrophia facialis [4]. This is characterized by thickening of the skin and underlying tissues by abnormally increased collagen deposition resulting in hardened lesions. PRS, a rare type of LS, is characterized by facial atrophy affecting the soft tissue and muscles as well as adjacent structures. PRS is categorized as mild, moderate, or severe on the basis of bone involvement in the region of the trigeminal nerve. The prevalence rate of PRS in general population is estimated to be at least 1 per 700,000 [5].

Soft-tissue facial asymmetry is rarely reported in the field of oral and maxillofacial surgery (OMS) group. Herein then, the case of a 27-year-old female patient with facial asymmetry and LS diagnosis is presented. We proposed that soft and hard tissue analyses for facial asymmetry were needed to allow effective, accurate, and beneficial treatment.

## Case presentation

A young female patient, aged 27 years, visited the hospital on November 18, 2009 complaining of facial asymmetry with right temporomandibular joint (TMJ) pain and noise, spasm of the right masseter muscle (MA), and trismus. Her mouth-opening range between the upper and lower incisors was 34 mm. Based on an analysis of the magnetic resonance imaging (MRI) findings, anterior disk displacement with reduction was diagnosed.

**Fig. 1:**
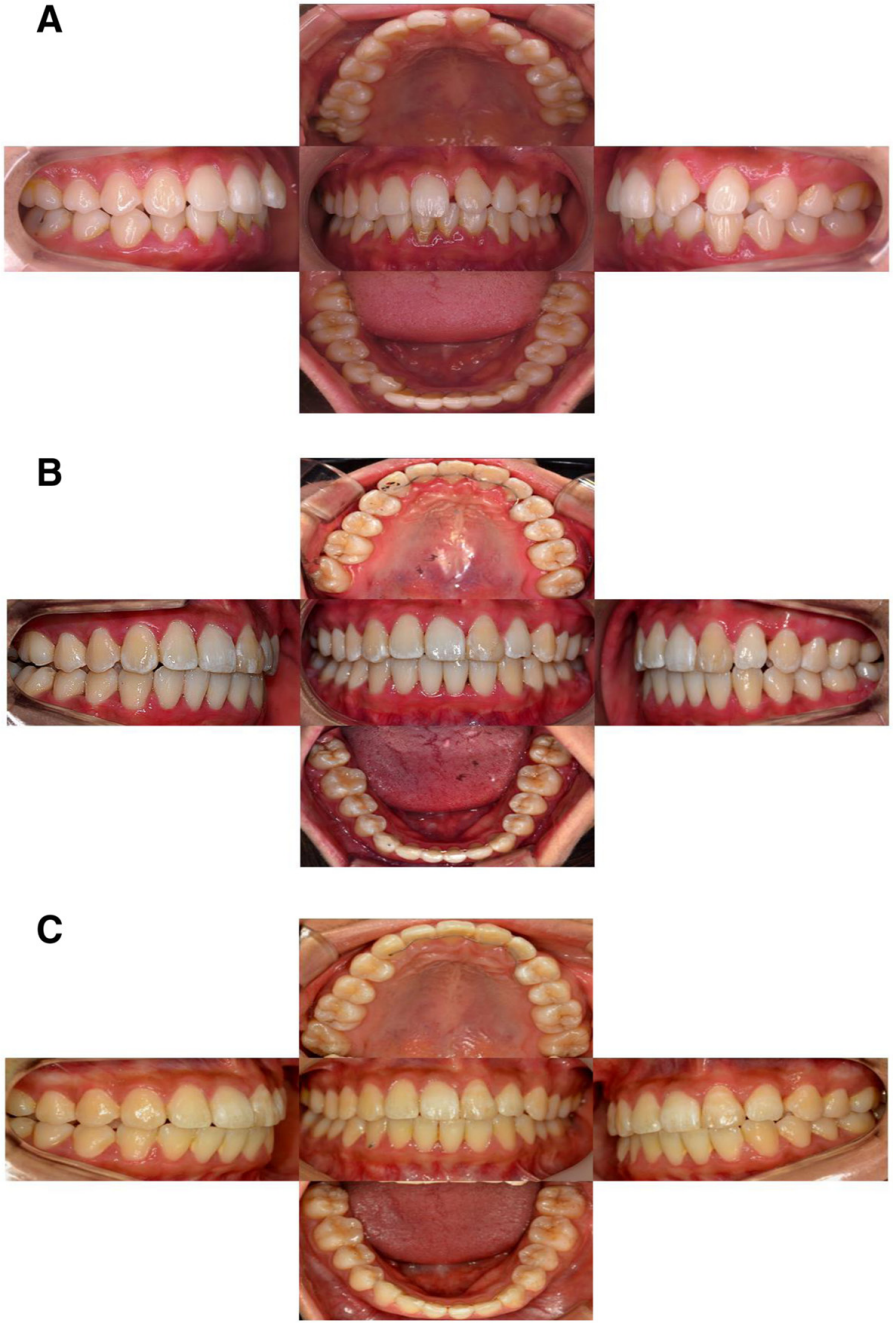
**a** Before orthodontic treatment. Intraoral photos were taken on 2 July 2010. **b** After orthodontic treatment. Intraoral photos were taken on 1 December 2012. **c** Follow-up check. Intraoral photos were taken on 13 December 2014

The patient also had right-side facial asymmetry. On June 9, 2010, she was evaluated by clinical examination, dental casts, and radiography. The clinical assessment involved visual inspection of the face and palpation to differentiate soft tissue and bone. Although the extra-oral examination found facial asymmetry with skin atrophy, the radiographic findings revealed no mandibular atrophy or deviation. The universal treatment plan entails the repair of bone defects via orthognathic surgery, though correction of limited soft-tissue facial asymmetry by this route is problematic. Therefore, we planned, for creation of an esthetically pleasing appearance, not orthognathic surgery but orthodontic treatment to level and align the dentition. Because she was afraid of the major surgery like orthognathic surgery. And we recommended, for correction of her facial asymmetry, if she so desired, autologous fat transplantation. The occlusion of the dentition was completed orthodontic treatment between November 2010 and December 2012 (Fig. 1a–c).

**Fig. 2:**
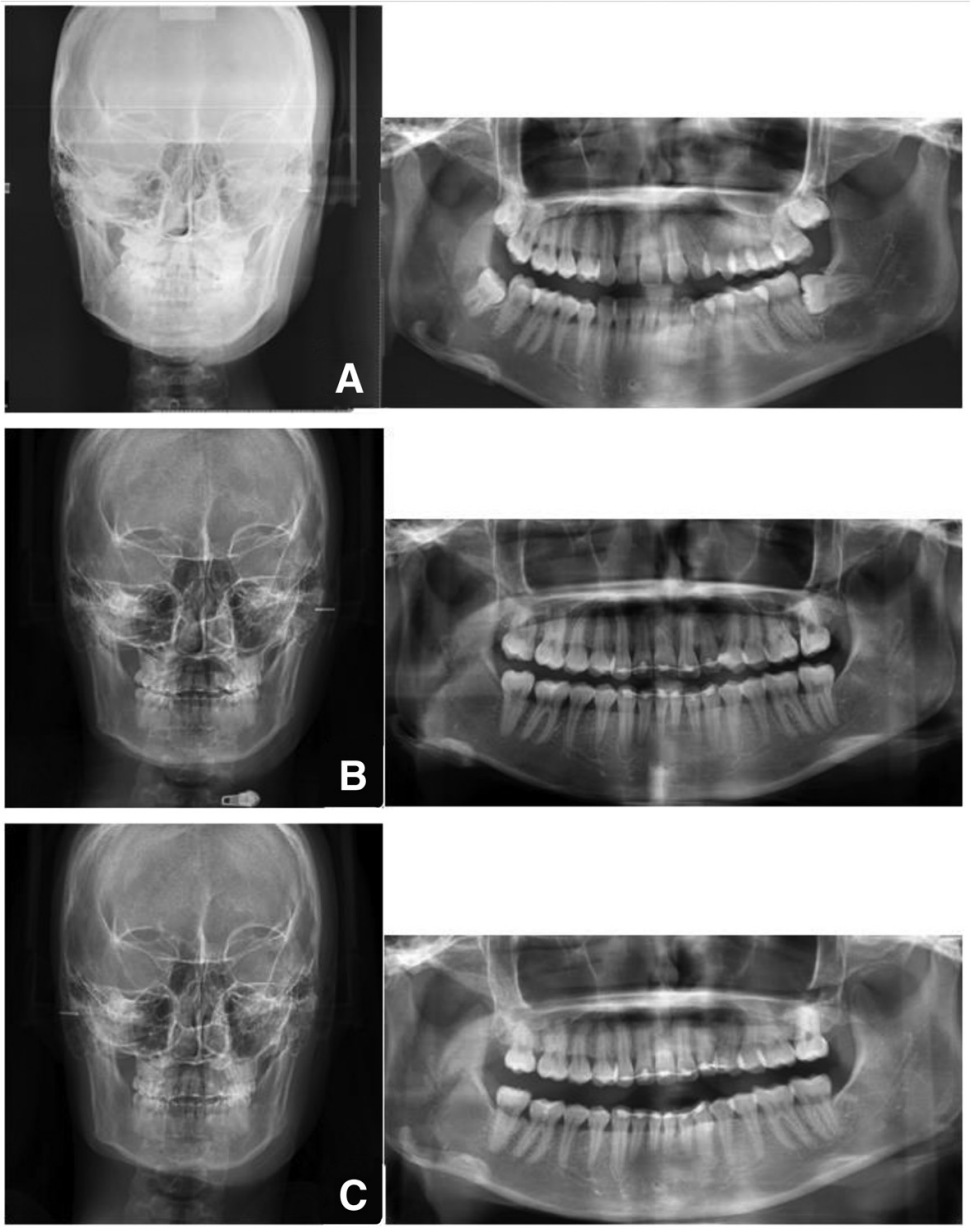
Radiographies were taken on **a** 2 July 2010, **b** 1 December 2012, and **c** 13 December 2014

The patient had no history of medical problems, trauma, and neurologic symptoms. During the clinical examination, we could not find any abnormal findings other than skin atrophy. Radiographically, atrophic soft tissue of the right chin and compensatory bone formation on the right mandible angle were observed (Fig. 2a–c). The general examination results, which included a complete blood cell count, serum chemistry, liver function tests, and chest radiography, were within the normal limits. Anti-nuclear antibody (ANA), anti-scl 70 antibody, anti-centromere B antibody, anti-ds DNA antibody, and rheumatoid factor (RF) tests also were all within normal ranges.

And we decided to reduce TMJ pain. The patient was treated for TMJ disorder (TMD) with occlusal stabilization appliance as well as arthrocentesis and lavage. Unilateral muscle spasm and trismus, additionally, were resolved by trigger point injection (TPI, 7:3 = dexamethasone disodium phosphate/lidocaine hydrochloride) and botulinum toxin therapy (Botox) of the right MA. After 5 months of treatment, she had no residual pain and boasted a painless TMJ range of motion and maximal 50 mm mouth-opening range. However, spasm of the right MA was observed once per week.

As muscle strain and spasm arose more frequently, we consulted the Rheumatoid Internal Medicine (RI) and Neurology (NU) departments for additional evaluation of the clinical findings, laboratory examination results, and radiographic imaging. On March 4, 2014, the patient was diagnosed, in RI, as LS with muscle spasm. At that time, she was treated by anti-gout and anti-inflammatory drugs and muscle relaxants. The administered therapeutic doses of medication were colchicine 0.6 mg/two times per day (b.i.d.), and eperisone 50 mg/b.i.d. for 4 months. The area of skin atrophy was softer in texture than before. Nevertheless, the patient often reported sudden onset of spasms of the right MA. Subsequently, she opted to discontinue treatment at RI and NU.

**Fig. 3:**
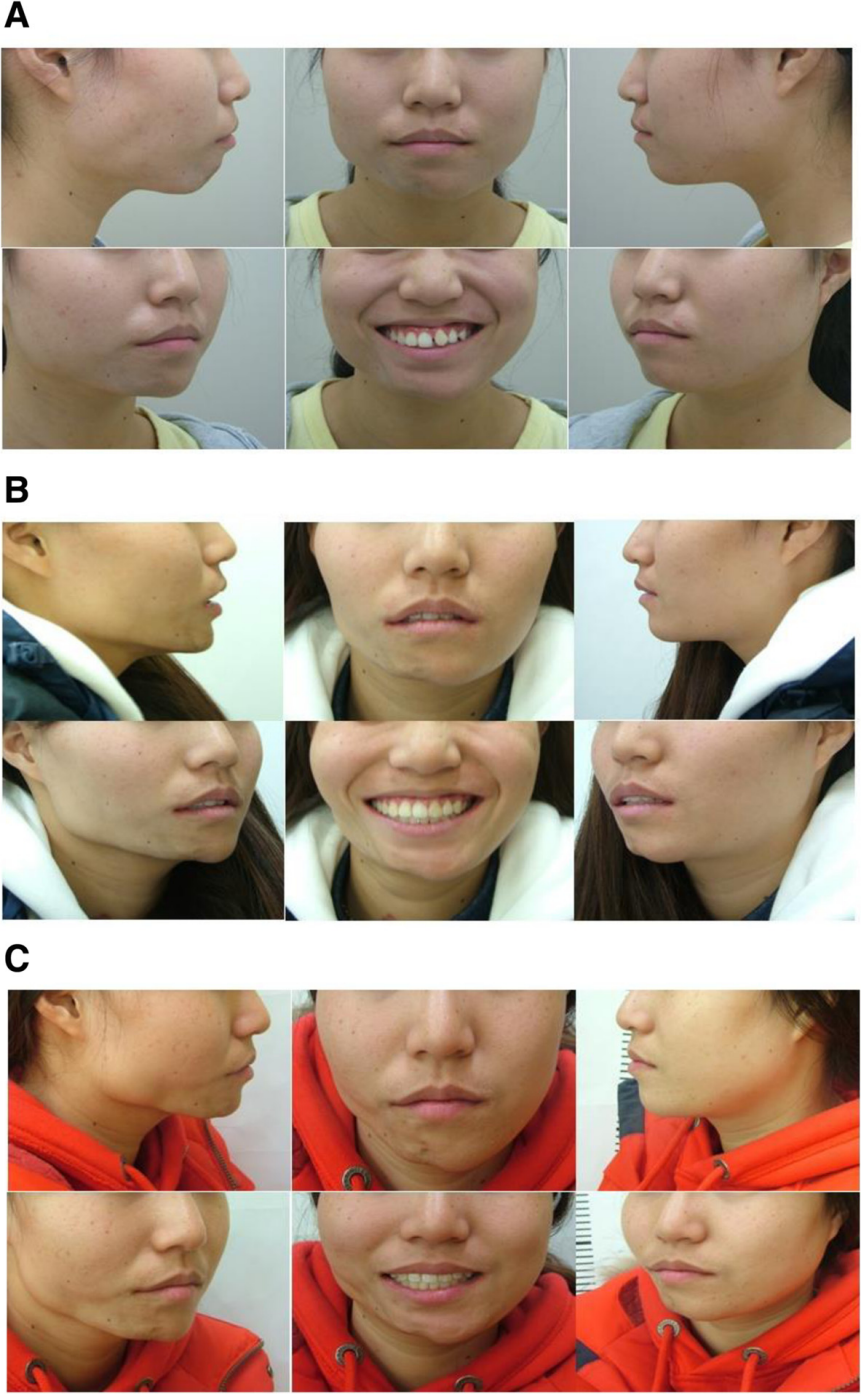
**a** Before orthodontic treatment. Extra-oral photos were taken on 2 July 2010. **b** After orthodontic treatment. Extra-oral photos were taken on 1 December 2012. **c** Follow-up check. Extra-oral photos were taken on 13 December 2014

Follow-up was carried out for 3 years, revealing good dentition; unfortunately, the patient’s soft-tissue facial asymmetry remained (Fig. 3a–c).

### Discussion

Facial asymmetry has dental, skeletal, and soft-tissue components. Its management entails a combination of orthodontic treatment and orthognathic surgery for bone deformities. Orthognathic surgery, however, is challenging, in that facial asymmetry treatment affects three-dimensional hard and soft tissue. Orthognathic surgery brings about a dermatic change, as soft tissue covers underlying bones. Limited soft-tissue asymmetry, meanwhile, is difficult to correct. The treatment modalities for the creation or restoration of an esthetically pleasing appearance were autogenous fat grafts, cartilage graft, and silicon injections.

LS has very diverse etiologies, and its pathogenetic mechanism is still unclear. It is an autoimmune disease characterized by cutaneous disorder and caused by increased collagen density resulting from a complex interplay of immune, genetic, extracellular matrix (ECM) disorder, and inflammation. Connective tissue disorders of the skin also show the characteristic activation of fibrosis resulting in excessive production and accumulation of collagen fibers. In its pathogenic features, LS has two main clinical categories that are recognized: systemic sclerosis (SSc), characterized by variable visceral involvement (mainly the esophagus, lung, and vascular system), and LS, which does not involve internal organs but is benign and self-limited (i.e., confined to the skin and/or subcutaneous tissues).

The common clinical signs of LS are linear scleroderma, plaque scleroderma, and generalized scleroderma. Cases of PRS, characterized by progressive hemiatrophy of the face and guttate scleroderma, are relatively infrequent.

Although the clinical aspects of scleroderma manifest at all ages, the peak age of incidence is the second decade of life [6]. LS more often occurs in women than in men, at a ratio of 3~4:1 [7]. Cho HK and Chun SI found that most patients are between 11 and 30 years of age and that women are affected more than men [6]. Kwon OS et al. reported a female-to-male ratio of 3.3:1 for morphea and of 4.1:1 for LS among those under age 30 [8]. Scrivener Y et al. concluded that 4 (7 %) of the 62 patients with LS shared clinical features on the cheek and/or mentum [9].

LS affects a variety of areas including the skin and adipose tissue as well as, sometimes, the fascia, muscles and bones [10]. Depending on the fibrotic action, which mainly affects the deeper layers of the connective tissue, LS rarely leads to esthetic problems, extremities atrophy, flexion contractures, and disability [11].

LS is differentiated from the systemic form of the disease, sclerosis, the Raynaud phenomenon (i.e., internal organ involvement). Diagnosis of LS, with respect at least to the proper identification of its distinct features, can be difficult. In 1995, Peterson LS et al. proposed an LS classificatory scheme that has since been adopted with few modifications by the majority of reviews published since that time (see Table 1) [12]. It groups LS into five subtypes: plaque morphea, generalized morphea, bullous morphea, linear morphea, and deep morphea. LS is diagnosed whenever a combination of two or more of these subtypes is present. In 2004, Pediatric Rheumatology European Society published a newly proposed LS classification scheme to correct that earlier put forward by Peterson et al.: circumscribed morphea, linear scleroderma, generalized morphea, pansclerotic morphea, and mixed morphea (see Table 2) [13]. In 2009, Kreuter A et al. suggested a subtype classification that considers the extent and depth of fibrosis and includes, correspondingly, the following four categories: the limited type, the generalized type, the linear type, and the deep type (see Table 3) [10].

**Table 1:** Classification of morphea or localized scleroderma

Plaque morphea	Plaque morphea
	Guttate morphea
	Atrophoderma of Pasini and Pierini
	Keloid morphea (nodular morphea) (Lichen sclerosus et atrophicus)
Generalized morphea	
Bullous morphea	
Linear morphea	Linear morphea (linear scleroderma)
	Morphea en coup de sabre
	Progressive facial hemiatrophy
Deep morphea	Morphea profunda
	Subcutaneous morphea
	Eosinophilic fasciitis
	Pansclerotic morphea of childhood

Source: Peterson LS et al., 1995 [12]

**Table 2:** Classification of morphea or localizes scleroderma

Circumscribed morphea	a) Superficial
	1. Deep
Linear scleroderma	a) Trunk/limbs
	1. Head
Generalized morphea	
Pansclerotic morphea	
Mixed morphea	

Source: Consensus Conference, Padua (Italy), 2004 [13]

**Table 3:** Classification of localized scleroderma

Limited type	Morphea (plaque type of localized scleroderma)
	Guttate morphea
	Atrophoderma idiopathica of Pasini and Pierini (superficial morphea)
Generalized type	Generalized localized scleroderma
	Disabling pansclerotic morphea
	Eosinophilic fasciitis (Shulman syndrome)
Linear type	Linear localized scleroderma of the extremities
	Linear localized scleroderma “en coup de sabre”
	Progressive facial hemiatrophy (Parry-Romberg syndrome)
Deep type	Deep morphea

Source: German guideline for the diagnosis and treatment of localized scleroderma, 2009 [10]

PRS is a rare disorder characterized by slow and progressive hemiatrophy of the soft tissue of the face, muscle, and bone. The etiologies of PRS are not well understood but are thought to include trauma, infection, and auto-immunity [14]. This syndrome involves dermatomes or multiple branches of the trigeminal nerve. It manifests as progressive shrinking and deformation of one side of the face, thus resulting in unilateral facial atrophy. The deformity effected by PRS is an issue not only of esthetics but also of facial functionality. PRS in fact has been linked with neurologic, ophthalmologic, rheumatologic, maxillofacial, and orthodontic issues [15].

Although PRS is often associated with linear scleroderma, differentiating it is very challenging. Moreover, either clinical symptom can slowly overlap with or transition into the other. There are no clear criteria universally agreed upon to differentiate PRS from linear scleroderma [16]; the clinical and laboratory features of both diseases have been found to coexist in many patients. However, the commonly reported neurologic manifestations of PRS are seizures and headaches [17].

Our patient did not show any neurologic lesion, though she did show clear features of facial asymmetry. Therefore, we diagnosed LS with right facial atrophy.

Although no effective global treatment for LS exists, a variety of therapeutic management modalities are available, among which are topical medications, immunosuppressive pharmacological agents, physical therapy, and phototherapy. The medication options include corticosteroids, methotrexate, calcipotriol, D-penicillamine, interferon-gamma, imiquimod, and tacrolimus. Unfortunately however, for some LS types, such treatment is of only limited effectiveness, and for some other LS types, symptoms worsen, or the risk of relapse is even higher, when so treated. The first case of successful ultraviolet radiation (UV) phototherapy for LS was reported in 1994. Since then, the literature on the entire spectrum of the anti-fibrotic and anti-inflammatory effects of psoralen plus UVA (PUVA), UVA-1, and topical photodynamic therapy has rapidly expanded [18]. In 2006, Lim SH et al. reported that low-dose UVA-1 was the phototherapy with the greatest efficacy and safety for LS patients [17].

There are many surgical-reconstructive techniques used in cases where there is loss of volume to the face or other area. Balaji SM proposed gluteal- and abdominal-fat grafting for PRS, by which, after the graft is harvested from the area near the inguinal crease, its tags are inserted into the atrophic lesion [19]. Zanelato TP et al., similarly, reported the implantation of autologous fat globules for PRS [20].

Therefore, we report the rare case of a facial asymmetry patient diagnosed as LS with soft-tissue atrophy. Clinically, our goal was to report on this soft-tissue facial asymmetry patient and to carefully diagnose, both preparatorily for surgery and to reduce diagnostic mistakes.

## Conclusions

In conclusion, oral and maxillofacial surgeons require, following their analysis of facial asymmetry patients, a treatment plan. It is important in this regard to differentiate limited soft-tissue facial asymmetry (without bone deformities) and bone-facial asymmetry. Facial asymmetry patients with skeletal asymmetry can be esthetically satisfied by orthognathic surgery; however, facial atrophy patients with skin or subdermal tissue contraction need, as in the present case, treatment by cosmetic dermatological surgery and orthodontic correction. We propose therefore the highlighting of this rare case in order to improve awareness and, especially, knowledge.

## Consent

Written informed consent was obtained from the patient for the publication of this report and any accompanying images.

## Abbreviations

PRS: Parry-Romberg syndrome
LS: localized scleroderma
OMS: oral and maxillofacial surgery
TMJ: temporomandibular joint
MA: masseter muscle
UV: ultraviolet radiation
